# Supplementary material for: The association between lead exposure and crime: A systematic review
Source: PLOS Glob Public Health. 2023 Aug 1;3(8):e0002177. doi: 10.1371/journal.pgph.0002177 (PMC10393136; doi:10.1371/journal.pgph.0002177)
Supplement: S1 Text — (DOCX) [file pgph.0002177.s001.docx]

**S1 Text**

**ProQuest Environmental Science Database:**

 "Lead"[Mesh] OR "Blood lead" OR "Lead Exposure" OR “Lead Exposure, Nervous System” [Mesh] OR "Lead Toxicity" OR "Blood lead levels” OR "Lead Poisoning" [Mesh] AND ("Crime" OR "Criminal behavior" OR "Convictions" OR "Arrests" OR " Delinquency" OR "Violent" OR "Violence" OR "Aggressiveness" OR "Violent Behavior" OR "Aggressive Behavior")

**PubMed:**  "Lead"[Mesh] OR "Blood lead" OR "Lead Exposure" OR “Lead Exposure, Nervous System” [Mesh] OR "Lead Toxicity" OR "Blood lead levels” OR "Lead Poisoning" [Mesh] AND ("Crime" OR "Criminal behavior" OR "Convictions" OR "Arrests" OR " Delinquency" OR "Violent" OR "Violence" OR "Aggressiveness" OR "Violent Behavior" OR "Aggressive Behavior")

**ToxNet:** "Lead"[Mesh] OR "Blood lead" OR "Lead Exposure" OR “Lead Exposure, Nervous System” [Mesh] OR "Lead Toxicity" OR "Blood lead levels” OR "Lead Poisoning" [Mesh] AND ("Crime" OR "Criminal behavior" OR "Convictions" OR "Arrests" OR " Delinquency" OR "Violent" OR "Violence" OR "Aggressiveness" OR "Violent Behavior" OR "Aggressive Behavior")

**Public Affairs Information Service (PAIS):**

( "Lead" OR "Blood lead" OR "Lead Exposure" OR “Lead Exposure, Nervous System”  OR "Lead Toxicity" OR "Blood lead levels" OR "Lead Poisoning" ) AND ("Crime" OR "Criminal behavior" OR "Convictions" OR "Arrests" OR " Delinquency" OR "Violent" OR "Violence" OR "Aggressiveness" OR "Violent Behavior" OR "Aggressive Behavior")
