# Supplementary material for: The association between lead exposure and crime: A systematic review
Source: PLOS Glob Public Health. 2023 Aug 1;3(8):e0002177. doi: 10.1371/journal.pgph.0002177 (PMC10393136; doi:10.1371/journal.pgph.0002177)
Supplement: S2 Table — (DOCX) [file pgph.0002177.s003.docx]

**S2 Table**

**Description of the Adapted ROBINS-E Framework for assessing risk of bias in environmental health studies for cross-sectional and case-control studies.**

|  | **Confounding Bias** | **Bias in participant selection** | **Exposure classification bias** | **Missing data bias** | **Outcome Measurement Bias** | **Bias in the selection of the reported result** |
| --- | --- | --- | --- | --- | --- | --- |
| Low | The authors controlled for socioeconomic status (SES), race (when needed), and a measure of home environment (proxy measures acceptable for SES and home environment) at minimum and all confounders were measured in a manner determined to be both reliable and valid. Additionally, the authors did not control for post-exposure variables that were affected by the exposure (i.e., no evidence of over-adjustment). | **If cross-sectional:** Sample must be representative of the population as a whole, sample size must be sufficiently large, and there must be evidence that appropriate inclusion/exclusion criteria were established within the study design phase.  **If case-control:**  Controls are representative of the population the cases are drawn from, loss to follow-up is not related to outcome or exposure status, self-selection bias is not related to exposure or disease, and there is no differential referral of subjects. | Well-defined exposure status. Robust, valid exposure assessment methodologies. Blood (venous), bone, or dentine lead levels used. Minimal evidence of differential misclassification of exposure.  Presence of minimal non-differential errors in exposure measurements. | No missing data **or** missing data was accounted for utilizing appropriate statistical methods. If complete case analysis, exclusion from the analysis was not likely to be related to the true value of the outcome. | Sensitive outcome measure, with outcome assessors blinded to exposure status, outcome assessment methods similar across groups, and all systematic measurement errors unrelated to the exposure received.  **If cross-sectional:** non-response must be accounted for and must be non-differential in nature. | The reported effect estimate was **not** likely to be selected from multiple outcome measurements or multiple exposure measurements. No evidence of the reported effect estimate coming from multiple analyses of the exposure-outcome relationship. |
| Some Concern | Potentially inappropriate statistical methodologies utilized to account for confounders **or** exclusion of one of the confounders listed above with inclusion of other relevant confounders. No evidence of over-adjustment present. All confounders are measured in a manner determined to be both reliable and valid. | **If cross-sectional:** Sample is representative of the population as a whole but the sample size is not sufficiently large. There must be evidence that appropriate inclusion/exclusion criteria were established within the study design phase.  **If case-control:** Controls are representative of the population the cases are drawn from, loss to follow-up is not related to outcome or exposure status **but** differential referral is present, or self-selection bias is present. | Well-defined exposure status. Blood (venous), bone, or dentine lead levels used. Evidence of differential misclassification of exposure **or** presence of non-differential errors in exposure measurements. **or** utilization of a less-robust exposure assessment methodology than the gold standard assessment strategies (i.e., capillary blood lead levels, single dentine measurement, issues of temporality). | Exclusion of participants due to missing data on exposure status **or** outcome status with no statistical methods employed to account for this missingness. If complete case analysis, exclusion from the analysis was not likely to be related to the true value of the outcome. | Sensitive outcome measure, with blinding of outcome assessors to exposure status probable but not explicitly stated, outcome assessment methods similar across groups, and all systematic measurement errors unrelated to the exposure received.  **If cross-sectional:** non-response must be non-differential in nature but is not fully accounted for. | The reported effect estimate was **not** likely to be selected from multiple outcome measurements but may be a result of multiple exposure measurements. No evidence of the reported effect estimate coming from multiple analyses of the exposure-outcome relationship. |
| High Risk of Bias | Missing or incomplete list of confounders **or** inadequate description of confounders (i.e., “all relevant confounders were adjusted for…” with no further information provided) **or** evidence of over-adjustment present/ invalid or unreliable measurement of confounders. | **If cross-sectional:** Sample is not representative of the population, and sample size is not sufficiently large. There must be evidence that appropriate inclusion/exclusion criteria were established within the study design phase.  **If case-control:** Controls are representative of the population the cases are drawn from, **but** loss to follow-up is related to outcome or exposure status and differential referral is present or self-selection bias is present. | Poorly defined exposure status with exposure assessment methods that are not robust, **or** poorly defined outcome parameters **or** evidence of substantial differential or non-differential errors in exposure measurement | Exclusion of participants due to missing data on exposure status **or** outcome status **or** confounding, with no statistical methods employed to account for this missingness. | Insensitive outcome measure, with no blinding of outcome assessors and outcome assessment methods similar across groups.  **If cross-sectional:** No evidence that non-response is non-differential in nature and no evidence that non-response is accounted for. | The reported effect estimate was likely selected from multiple outcome measurements **and/or** multiple exposure measurements. No evidence of the reported effect estimate coming from multiple analyses of the exposure-outcome relationship. |
| Very High Risk of Bias | No appropriate analysis method utilized to adjust for critically important confounders or a failure to account for confounding. | **If cross-sectional:** Sample is not representative of the population as a whole, sample size is not sufficiently large, and there is no evidence that appropriate inclusion/exclusion criteria were established within the study design phase.  **If case-control:** Controls are **not** representative of the population the cases are drawn from, **and/or** loss to follow-up is related to outcome or exposure status **and/or** differential referral is present, or self-selection bias is present. | Poorly defined exposure status with exposure assessment methods that are not robust **and** evidence of substantial differential or non-differential errors in exposure measurement. | Exclusion of participants due to missing data on exposure status **and** outcome status with no statistical methods employed to account for this missingness **and/or** a discontinuity in the proportion of participants excluded or the reasons for missingness across exposure status. | Insensitive outcome measure, lack of blinding, varying outcome assessment methods across groups, and systematic measurement errors likely related to the exposure received.  **If cross-sectional: No critical criteria, serious is last category for outcome measurement bias.** | The reported effect estimate was likely to be selected from multiple outcome measurements within the outcome and exposure domain and multiple analyses of the exposure-outcome relationship within different subgroups. |
