# Supplementary material for: The association between lead exposure and crime: A systematic review
Source: PLOS Glob Public Health. 2023 Aug 1;3(8):e0002177. doi: 10.1371/journal.pgph.0002177 (PMC10393136; doi:10.1371/journal.pgph.0002177)
Supplement: S3 Table — (DOCX) [file pgph.0002177.s004.docx]

**S3 Table**

**Justification for Risk of Bias Designations**

| Study Author | Confounding Bias | Selection Bias | Exposure | Missing Data | Outcome | Reporting | Conflict of Interest |
| --- | --- | --- | --- | --- | --- | --- | --- |
| Aizer & Currie, 2019 | Included race (needed), socioeconomic status or a proxy, and a measurement of home environment or a proxy | Selection into the study is **not** related to both exposure and outcome. | Exposure was measured using a combination of capillary and venous blood, with no discrete measurements of either | Missing data well accounted for | Data on detentions are available for only the years of  2004 to 2014 despite RI having no min. age for adult prosecution. | The reported effect estimate was **not** likely to be selected from multiple outcome measurements or multiple exposure measurements. No evidence of the reported effect estimate coming from multiple analyses of the exposure-outcome relationship. | No discussion on COI |
| Beckley et al., 2017 | Missing race (needed) and measure of home environment | Prospective cohort | Blinded, well-defined exposure status with robust measurement methodologies. | Missing outcome and exposure data, however the authors appropriately discuss and account for this | Blinding presumed but not explicitly stated | The reported effect estimate was **not** likely to be selected from multiple outcome measurements or multiple exposure measurements. No evidence of the reported effect estimate coming from multiple analyses of the exposure-outcome relationship. | No conflict of interest reported |
| Dietrich et al., 2001 | Included race (needed), socioeconomic status or a proxy, and a measurement of home environment or a proxy | Prospective cohort | Blinded, well-defined exposure status with robust measurement methodologies | Missing data on Pb measurements with no statistical analysis to account for this | It is assumed that the staff who completed interviews were blinded but never explicitly stated | The reported effect estimate was **not** likely to be selected from multiple outcome measurements or multiple exposure measurements. No evidence of the reported effect estimate coming from multiple analyses of the exposure-outcome relationship. | Conflict of interest reported, not concerning |
| Emer et al., 2020 | No measurement of home environment or a proxy | Selection into the study is **not** related to both exposure and outcome. | Utilized capillary samples, a less accurate measurement modality for lead | Missing data on exposure with no discussion of why or statistical analysis to account for this | Outcomes are classified in an ambiguous manner (see Discussion Section) | The reported effect estimate was **not** likely to be selected from multiple outcome measurements or multiple exposure measurements. No evidence of the reported effect estimate coming from multiple analyses of the exposure-outcome relationship. | No conflict of interest reported |
| Fergusson et al., 2008 | Included race (needed), socioeconomic status or a proxy, and a measurement of home environment or a proxy | Prospective cohort | Exposure (dentine blood lead) was well-defined prior to assessing outcome, however only two measurements from a single tooth were used, making these methods less robust than the multiple BLL’s obtained in other studies. | Appears to be no missing data from the sample | Blinding assumed but not explicitly stated | The reported effect estimate was **not** likely to be selected from multiple outcome measurements or multiple exposure measurements. No evidence of the reported effect estimate coming from multiple analyses of the exposure-outcome relationship. | No conflict of interest reported |
| Naicker et al., 2018 | Missing race (not needed) home environment | Sample size is not sufficiently large, study population is not representative of the population at large; however, exclusion/inclusion criteria are clearly listed. | Utilized capillary samples, a less accurate measurement modality for lead | Missing data on both outcome and exposure with no discussion on how to account for that data. | Self-reported questionnaires with no administrative assistance from trained staff; high concern for inaccuracies | The reported effect estimate was **not** likely to be selected from multiple outcome measurements or multiple exposure measurements. No evidence of the reported effect estimate coming from multiple analyses of the exposure-outcome relationship. | No discussion on COI |
| Needleman et al., 1996 | Included socioeconomic status or a proxy, and a measurement of home environment or a proxy, but did not include race (needed). | Those subjects at highest risk for delinquency were selected with no discussion on how they may fundamentally differ from those not selected in the risk of both exposure and outcome | Exposure measured via x-ray; blinding assumed but not reported | Presence of potential differential misclassification in exposure and potentially in outcome | Blinding probable but not explicitly stated. | The reported effect estimate was **not** likely to be selected from multiple outcome measurements or multiple exposure measurements. No evidence of the reported effect estimate coming from multiple analyses of the exposure-outcome relationship. | No discussion on COI |
| Needleman et al., 2002 | Included race (needed), socioeconomic status or a proxy, and a measurement of home environment or a proxy | Loss to follow up likely related to both exposure and outcome. Self-selection bias present. | Exposure measured via x-ray; blinding assumed but not reported | Large potential discrepancies in baseline characteristics and probability of experiencing exposure and outcome between groups designated as cases/non cases and controls/non controls | Blinding probable but not explicitly stated | The reported effect estimate was **not** likely to be selected from multiple outcome measurements or multiple exposure measurements. No evidence of the reported effect estimate coming from multiple analyses of the exposure-outcome relationship. | No conflict of interest reported |
| Nkomo et al., 2017 | Included race (not needed), socioeconomic status or a proxy, and a measurement of home environment or a proxy | Longitudinal birth cohort | Blinding not explicitly stated | None | Outcome measurement is reliable and validated | The reported effect estimate was **not** likely to be selected from multiple outcome measurements or multiple exposure measurements. No evidence of the reported effect estimate coming from multiple analyses of the exposure-outcome relationship. | No discussion on COI |
| Nkomo et al., 2018 | Included race (not needed), socioeconomic status or a proxy, and a measurement of home environment or a proxy | Longitudinal birth cohort | Blinding not explicitly stated | None | Blinding not explicitly stated | The reported effect estimate was **not** likely to be selected from multiple outcome measurements or multiple exposure measurements. No evidence of the reported effect estimate coming from multiple analyses of the exposure-outcome relationship. | No discussion on COI |
| Olympio et al., 2010 | Missing race (not needed) a measurement of home environment | The choice of study participants indicates that this cross-sectional study may not be representative of its underlying population. | Blinding not explicitly stated | Only 55% of the total enrolled subjects were examined for lead and filled out the SRD form, and of the 45% of parents who answered the CBCL, only 93 children were examined for lead. No discussion on handling of missing data. | Measures of outcome for parental self-report are poor; the interviewees had to verbally respond to personal questions about their child’s behavioral characteristics – this is an insensitive measure and blinding is not mentioned. | The reported effect estimate was **not** likely to be selected from multiple outcome measurements or multiple exposure measurements. No evidence of the reported effect estimate coming from multiple analyses of the exposure-outcome relationship. | No conflict of interest reported |
| Renzetti et al., 2022 | Included race (not needed), socioeconomic status or a proxy, and a measurement of home environment or a proxy | Sample size was sufficiently large, with appropriate inclusion/exclusion criteria applied | Blinding not explicitly stated | No missingness reported | Blinding not explicitly stated, however non-response was not an issue in this study and the outcome measurement was sensitive. | The reported effect estimate was **not** likely to be selected from multiple outcome measurements or multiple exposure measurements. No evidence of the reported effect estimate coming from multiple analyses of the exposure-outcome relationship. | No discussion on COI |
| Sampson & Winter, 2018 | Included race (needed), socioeconomic status or a proxy, and a measurement of home environment or a proxy | Selection into the study is **not** related to both exposure and outcome. | Measurement error was reduced by using an average BLL across test results. Blinding not explicitly stated | Of the 378 eligible birth cohort members, 254 were matched with blood lead levels, however selection into the sample was adjusted for using the CDPH lead testing coverage rate in each child’s neighborhood. Analyses were restricted to complete information on all measures, thus the primary sample had only 212 individuals. | No blinding although it can be presumed from study design. Validity check performed on the CBCL; arrest history was conducting using a matching process that captured the time period well before the age of first arrest and prevented any censoring of criminal history. | The reported effect estimate was **not** likely to be selected from multiple outcome measurements or multiple exposure measurements. No evidence of the reported effect estimate coming from multiple analyses of the exposure-outcome relationship. | No discussion on COI |
| Thomson et al., 1989 | Incomplete list of confounders | Study authors give no information on the differences in characteristics between the top sub-sample (top quartile of lead exposure) (n=501) and the remainder of the study population (n=855).  It is likely that variables that influenced eligibility selection were influenced by the outcome. | Exposure methods are not well discussed, i.e., was there adequate quality control, what were laboratory standards | When there were missing items on the parents’ ratings, they were simply scored as zero. There is no report of how much data was ultimately missing from the ratings scores. | Outcome measurement is sensitive, however missing outcome data is not reported on and there is no information about whether those missing outcomes are related to the exposure received. | The reported effect estimate was likely selected from multiple outcome measurements within the outcome domain. The stepwise regression reporting modality left the definition of included covariates unclear, and exact values for the log-odds are not reported. | Clearly disclosed; not of concern. |
| Tlotleng et al., 2022 | Included race (not needed), socioeconomic status or a proxy, and a measurement of home environment or a proxy | Longitudinal birth cohort | Blinding not explicitly stated; well defined exposure status with repeated measurements for accuracy. | No missingness observed | Blinding not explicitly stated | The reported effect estimate was **not** likely to be selected from multiple outcome measurements or multiple exposure measurements. No evidence of the reported effect estimate coming from multiple analyses of the exposure-outcome relationship. | No discussion on COI |
| Wright et al., 2008 | Included socioeconomic status or a proxy, and a measurement of home environment or a proxy, but did not include race (needed). | Participants were drawn from a prospective birth cohort and the participants within the analysis were not substantially different from those with missing data at baseline. | Well-defined exposure status with the outcome clearly defined prior to outcome assessment. | There is missing exposure data, but it was appropriately imputed to avoid excluding participants unnecessarily. Measures of BLL were collected over an average of many years, and 89-92% of the cohort had a full range of blood samples from 3 mo-5 years of age. Potential for missing data on outcome d/t delay in court reporting. | Measurement of outcome was slightly insensitive as the courts in Hamilton Co., OH, do not regularly update their arrest records, leaving a subset of the arrested population potentially missing from analysis. | The reported effect estimate was **not** likely to be selected from multiple outcome measurements or multiple exposure measurements. No evidence of the reported effect estimate coming from multiple analyses of the exposure-outcome relationship. | No conflict of interest reported |
| Wright et al., 2021 | Included socioeconomic status or a proxy, and a measurement of home environment or a proxy, but did not include race (needed). | Participants were drawn from a prospective birth cohort and the participants within the analysis were not substantially different from those with missing data at baseline. | Well-defined exposure status with the outcome clearly defined prior to outcome assessment. | Authors state that the cohort was examined to assess for any significant differences in blood lead levels or developmental indices due to missing data. No significant differences were found, and there was no evidence of nonrandom attrition due to death or incarceration | Measurement of outcome was slightly insensitive as the courts in Hamilton Co., OH, do not regularly update their arrest records, leaving a subset of the arrested population potentially missing from analysis. | The reported effect The reported effect estimate was **not** likely to be selected from multiple outcome measurements or multiple exposure measurements. No evidence of the reported effect estimate coming from multiple analyses of the exposure-outcome relationship. | No conflict of interest reported |
