# Supplementary material for: The association between lead exposure and crime: A systematic review
Source: PLOS Glob Public Health. 2023 Aug 1;3(8):e0002177. doi: 10.1371/journal.pgph.0002177 (PMC10393136; doi:10.1371/journal.pgph.0002177)
Supplement: S4 Table — (DOCX) [file pgph.0002177.s005.docx]

**S4 Table**

**Variation in Confounders by Study Author**

| Study Authors | List of Confounders |
| --- | --- |
| Aizer & Currie, 2019 | Socioeconomic status measured via free lunch status, year of birth, municipality, gender, race, ethnicity, maternal age, maternal education, marital status, child’s birth order, birthweight |
| Beckley et al., 2017 | Socioeconomic status, sex of child *Note: all lead levels were corrected for hematocrit, but hematocrit was not included as a confounder* |
| Dietrich et al., 2001 | Socioeconomic status (current), mean HOME score*, parental IQ, and birth weight |
| Emer et al., 2020 | Socioeconomic status, sex of child, race, year of birth |
| Fergusson et al., 2008 | Socioeconomic status measured by maternal education and child's ethnicity (Maori or non-Maori), Family Functioning as measured by family conflict, maternal smoking during pregnancy, exposure to childhood physical abuse, parental alcohol abuse or disuse, and parental offending, and Intervening Factors such as grade point average and leaving school without qualifications (i.e., the equivalent of a GED or high school diploma in the United States) |
| Naiker et al., 2018 | Income, employment, education level, and frequency of recreational shooting |
| Needleman et al., 1996 | Socioeconomic status measured via maternal occupation, maternal education, and maternal IQ, Family Function measured via the presence of one versus two parents in the home, mothers' age at subjects' birth, and the number of children in the family; race, history of medical problems. |
| Needleman et al., 2002 | Parental education, parental occupation (measured via Hollingshead score**), race, presence of one or two parents in the home, and neighborhood crime rate |
| Nkomo et al., 2017 | Socioeconomic status categorized by the presence or absence of various household items, gender, maternal education at birth, maternal marital status, maternal age at birth, residential area of birth |
| Nkomo et al., 2018 | Socioeconomic status categorized by the presence or absence of household items, gender, maternal education at birth, maternal marital status, maternal age at birth, residential area of birth |
| Olympio et al., 2010 | Maternal education level, occupation of the head of household, number of children and number of total individuals living in the house, age and sex of the adolescent, and whether or not the adolescent's parents were living together or not at the time of the study |
| Renzetti et al., 2022 | Sex, age, SES, distance from the exposure source, child IQ, exposure to passive smoking and the total HOME score |
| Sampson & Winter, 2018 | Poverty as measured by the TANF ***, sex, race/ethnicity, immigrant generational status of primary caregiver, marital status of primary caregiver, education level of primary caregiver, age of child, and a measure of neighborhood racial composition and poverty |
| Thomson et al., 1996 | 30 variables included as confounders, but complete list is either unavailable or variables are undefined. Defined variables include sex and length of gestation. Undefined variables include family structure, parents' participation with child, parent/child communication, and parents' matrices test. |
| Tlotleng et al., 2022 | Age, sex, level of schooling (categorized into three: Grade 5 or less, grade 6–12 and tertiary education), presence of both parents at home, home environment, neighborhood crime, profile of illegal substance abuse, use of alcohol and socio-economic factors (maternal education, type of housing and occupation status). |
| Wright et al., 2008 | Socioeconomic status measured via Hollingshead score**, sex, mean HOME score*, birth weight, maternal smoking during pregnancy, maternal alcohol use, maternal marijuana and narcotic use, total prior maternal arrests, number of children in the home, and whether the mother was on public assistance during the participant's childhood. |
| Wright et al., 2021 | Birth weight (grams), maternal age at delivery, APGAR scores at 1-minute post-birth, self-reported maternal alcohol, marijuana, and tobacco use, maternal IQ measured by the WAIS-R**** |

## **HOME score (Home Observation Measurement of the Environment) is a measure of the quality of a child's home environment **Hollingshead score, a combined measure of occupation, education, sex, and marital status ***TANF is the Temporary assistance for needy families program ****WAIS-R is the Wechsler Adult Intelligence Scale-Revised*
