# Supplementary material for: The association between lead exposure and crime: A systematic review
Source: PLOS Glob Public Health. 2023 Aug 1;3(8):e0002177. doi: 10.1371/journal.pgph.0002177 (PMC10393136; doi:10.1371/journal.pgph.0002177)
Supplement: Attachment — Submitted filename: Response to Reviewers.pdf [file pgph.0002177.s006.pdf]

Milken Institute School of Public Health
THE GEORGE WASHINGTON UNIVERSITY

Dr. Maria Jose Talayero Schettino
Department of Environmental and Occupational Health
The George Washington University
950 New Hampshire Avenue
Washington, DC, 20052
(917) 972-4571
mjtalayero@gwmail.gwu.edu

Dr. Naveen Puttaswamy, Ph.D., Academic Editor
Dr. Jonathan Huang and Associates, Peer Reviewers
Journal of PLOS Global Public Health
1265 Battery Street, Suite 200
San Francisco, CA
94111 USA
(415) 624-1200
em@editorialmanager.com

April 21, 2023

Dear Drs. Puttaswamy, Huang, and Associates,

Thank you for your letter regarding our manuscript entitled “The association between lead exposure and crime: a systematic review.” We appreciate your consideration and time regarding this manuscript, and we think you will find that the revised and updated version is appropriate for publication in *PLOS Global Public Health.*

Our team thoroughly reviewed each comment, and we are grateful for such detailed and thoughtful feedback. We have attached a detailed table to this letter with each individual comment addressed, excepting those discussed here. The majority of the suggestions and corrections made by the reviewers were implemented into the latest draft.

A few points were brought up by reviewers that merited discussion here. One of the concerns expressed across multiple reviewers involved our classification of the prenatal and early childhood window as statistically significant, peak windows of exposure. As much of this data did arise from a single cohort, we understand the concern and as such, have modified our manuscript to remove this conclusion. We have also altered our discussion to include a deeper, more balanced discussion of the trends seen across the evidence, particularly in relation to the ROBINS-E tool.

Two reviewers expressed concern that neighborhood factors were not more deeply discussed in the manuscript or listed as critical confounders. We included socioeconomic status and home environment as critical confounders. home environment was typically measured by a HOME score

or by a combination of HOME score and maternal IQ. HOME scores encompass a list of contextual environmental factors, and low HOME scores have been associated with later negative behavioral outcomes. We did not choose to include further neighborhood factors as critical confounders because we felt the home environment would yield a stronger contextual measure of risk.

We appreciate the comment regarding the paucity of studies from within Asia. Our review was conducted with the help of a librarian and encompassed all studies on a global scale with no language or geographic restriction that pertained to aggressive and criminal behaviors and lead exposures. We did not intentionally exclude studies from within Asia, there simply were none to analyze per the parameters of the review. In our updated draft, we have made a strong recommendation encouraging further research within this field on a more diversified, global scale.

We feel that the peer review process has added to the strength of our paper and look forward to your further feedback and review. Please do not hesitate to contact us if there are any further questions.

With thanks for your time and assistance,

Dr. Maria Jose Talayero

Reviewer	Comment	Line Addressed	Comment from authors
**Reviewer 1**	It is not clear why the authors emphasized the elimination of six major ecological studies. It does not affect the conclusions of the article, but it would be interesting to state the reasoning behind this decision, since it was highlighted in the description of the studies that were included or not included.	Lines 469 - 475	Thank you for pointing this out. Our reasoning is as follows: In ecological studies, the unit of observation is never a single individual but rather an entire population or a specific group of people usually living in a common area, or with common conditions. However, within that area, the individual conditions and exposures might vary enormously (for example, in a county with low socioeconomic status, you might have people with high level of education, company owners, living in wealthy conditions). In this case, as there is no distinct information regarding disease, risk, or outcome in an individual, it becomes impossible to individually link the potential exposure to the outcome, and just describe a correlation that is difficult to assess as causal. Such studies generate hypothesis to be confirmed with individually based studies. Our original research question was intended to review all available literature that covered individual-level lead exposures with outcomes relating to crime. The six studies excluded are studies that are well known and have been cited repeatedly within the literature, so we felt it was important to specifically state that they were excluded not because they were poorly designed, but rather simply because they covered population-level outcomes rather than individual-level outcomes. Additionally, PRISMA guidelines recommend a brief description of studies excluded, thus our

			decision to emphasize the elimination of these studies. A summary of this has been added to the text.
	The discussion of confounding variables is of particular relevance to this article and was well done. Mentioning it in the abstract could be beneficial for the reader.	Lines 35 -37	The inclusion of the discussion on confounding variables has now been added to the abstract.
**Reviewer 2**	This scientific work assessed the association between lead exposure and crime, my suggestion to the authors is to explain molecular mechanisms of the potentially hazardous effects of lead exposure.	Lines 70 - 77	Molecular mechanisms have been added to the Introduction
	Figure 1: The authors mentioned that the total studies excluded were 50, however, the summation of these studies is 49 (34+10+3+2). Please, correct that.	Figure 1. Prisma Flow Diagram	Thank you for pointing out this error. It has been corrected on Figure 1, PRISMA Flow Diagram.
	In lines 56 and 57, the authors raise a concern about lead exposure without mentioning the sources of exposure, can the authors give some details about the sources of exposure in developing and developed countries?	Lines 56 - 61	Sources of exposure are now found in the introduction.
	In line 65, the authors mentioned inconsistent results of the epidemiological studies and on page 28, some conflicting data regarding antisocial behaviour, can the authors add some more explanation to these inconsistencies?	Discussion Section	We have re-written the discussion section in a way that we hope adds some clarity to the question of antisocial behavior.
	Some technical problems occurred many times in the text, “Error! Bookmark not defined”. Can the authors resolve this technical issue?	N/A	Thanks for letting us know, on our end this does not show, but we have contacted the editors to make sure this does not happen on the reviewer’s end.
	The authors mentioned on page 35 that the explanation of the mechanisms of the potentially hazardous effects of low-level lead exposure are not fully understood. Can the authors add some of the published mechanisms?	Add published mechanisms - added under consistency with existing literature, 2 paragraphs in	In section “Consistency with existing literature”

	In the last sections the authors mentioned the confounders that may impact the association with criminal behaviours. Can the authors shed the light besides the socioeconomic status, the nutritional status of both mother, infants, and children and exposure to other environmental contaminants like other heavy metals and or pesticides which may impact this association?	N/A	Potential confounding variables that we considered but ultimately did not include for our risk of bias assessment were drawn from all of our included studies, and covered variables related to:<br>-Perinatal and child health factors<br>-Maternal age at birth of subject<br>-Birth weight<br>-Birth length<br>-Neonatal head circumference<br>-Cigarette consumption during pregnancy (1/2 packs/day)<br>-Alcohol consumption during pregnancy (yes/no)<br>-Marijuana consumption during pregnancy (yes/no)<br>-Narcotics use during pregnancy (yes/no)<br>-Child's iron status at 1– 6 years (hemoglobin, hematocrit and total iron<br>binding capacity)<br>-Consumption of alcohol and tobacco during adolescence<br><br>In regards to the reviewer's point about other heavy metals or pesticides that may confound this association - organophosphate pesticides (OP) in particular have been linked to a lower IQ and in select cases, aggression, however the association between OP and aggression is much weaker than the association between lead and aggression (Vernet et al., 2021). Many of the heavy metals (such as mercury and manganese) have been studied interacting with lead, for

			neurotoxic effects, attention deficit and behaviors, but they are not yet established as related to aggressive behavior. However, these metals are typically not used in widespread applications in the way lead is and tend to be geographically centered to particular regions or industries, thus the studies included in our review did not see fit to include these metals or pesticides (Haynes et al., 2011).
	The authors mentioned in table 2 the location where the previous studies were conducted and according to this table, many of these studies were conducted in the USA and no study was carried out in Asia. So, in the recommendation section, can the authors add a recommendation of conducting studies in other locations to better assess this relationship by putting into consideration gene-environments interaction and the difference in the social and economic determinates?	Lines 648-649	As demonstrated by Table 2, our literature search yielded studies predominantly conducted within the United States, South America, and Africa. We strongly recommend that further studies on the association between crime and lead be carried out in a more diverse range of countries in order to better assess the relationship between genetic factors and the living environment.
**Reviewer 4**	more could have been done to contextualize the state of the literature, particularly in relation to the recent meta-analyses by Higney, et al. they briefly mention. The previous study, conducted by (I believe) economists, give ample room for the current authors to do a more in depth evaluation of the individual-level studies identified here. For example, Higney, et al point out (it is mentioned here, too) that ecological studies showed substantially larger effect sizes than individual-level studies, with one possibility being, e.g. neighborhood confounding. Was there a discussion or assessment of neighborhood factors in the current set of studies? I was surprised that	N/A	Please see above for a deeper discussion of the neighborhood confounding question. The ROBINS-E tool does not evaluate publication bias within its ROB tool; thus we chose to refrain from a discussion on publication bias; however your point is well-taken - it is possible that publication bias may impact the observed significant associations demonstrated within the review. We had hoped to standardize all effect sizes, but due to the disparate nature of the hypothesis tests included, we were unable to do so. Regarding the point about effect sizes and

	contextual factors were not even mentioned in the list of critical confounders. Further, Higney, et al identified substantial publication bias. Was this evaluated here? Did the studies with smaller / negative effect sizes have stronger designs / less risk of bias? On that subject, given the use of ROBINS-E, I had hoped there would be greater discussion & comparisons regarding key features of the included studies to draw out patterns and make recommendations for future studies. Are certain exposure measurements and study designs more credible than others as experimental analogues? Which design/analytic features were related to smaller or larger effect sizes (or null findings). The use of nominal statistical significance (i.e. p-value thresholds) to compare findings, such as across ages of exposure, does not seem particularly relevant here, especially as sample sizes varied greatly. Standardized effect sizes would be much more useful (along with the estimates of variability). On that note, it was difficult to understand the various effect sizes presented as they were unit-less (how much of an increment in lead?) and varied between betas and IRRs. More stratification and comparisons of strength of evidence by risk of bias would be welcome.		whether or not that correlates with a stronger or weaker study design or larger sample size: we wanted to discuss this in depth, but were hindered by several things. There was so much disparity across included confounders, the measurement of said confounders, the classification of outcomes, the exposure metric used, the geographic regions assessed, the time windows of assessment, etc., that it was extremely difficult for us to accurately assess the credibility and strength of various study designs in comparison to other included studies. We hope this helps explain the lack of discussion in our paper on these topics.
	Finally, the authors rightfully mentioned that understanding mediators and moderators would strengthen the evidence for a causal link between lead and crime, particularly with respect to alternatives hypotheses regarding education, IQ, aggressive behaviors, opportunity, etc. Was there anything in the studies reviewed that specifically support or point in these directions? Do differences in effects across types of outcomes, e.g. violent vs. non-violent, point in a given direction? What about support for the hypothesis that age 6 is a critical window? What specifically should be looked at next to better understand	Lines 648-651	The studies we reviewed pointed to education, IQ, socioeconomic opportunity, and aggressive behaviors as potential mediators/moderators, with a detailed literature review and discussion given for each. We had hoped to specifically point our readers to differences in effect across violent vs non-violent outcomes but found that there was too much heterogeneity across outcome classification to adequately do so. It was from this literature that our recommendations for

	these factors?		moderation and mediation analyses were drawn from. We have removed the hypothesis that age 6 is a critical window and recommended further research on windows of exposure. We strongly suggest that future studies focus ample discussion on mediators and moderators of concern when evaluating these associations.
	-Table 4: Thomson et al is 1989 not 1996?	Table 4	This has been corrected in Table 4
	-In the Results, it would be helpful to see the units of each of the measures even if they cannot be harmonized	Table 3 and Results Section	Units are explicitly shown now on Table 3 and the Results section
	-Discussion, General Interpretation: "consistent with epidemiological research..." aren't all these studies reviewed epidemiologic research? Perhaps this should be rephrased to specifically discuss the critical period / life course models presented in the given citations (39,40)	Discussion Section, General Interpretation	This paragraph was rephrased
	-Trends in Evidence: Again it would be helpful to see a breakdown in the effect sizes / presence / absence of findings based on risk of bias or other design features -- Did any adjust for neighborhood characteristics? did those have smaller effect sizes?	Table 3<br>Line 561-565	ROB results are now shown in Table 3 to show study characteristics and ROB in the same place.<br>Neighborhood characteristics were assessed in some of the studies as confounders and included in the ROB assessment include: socioeconomic status and home environment.<br>One of the characteristics that was not assessed as a neighborhood characteristic in any study is cultural factors, which may play a critical role in lead exposure. This is the case for Latin American countries that use lead-glazed-clay ceramics as part of their culture. In countries such as Mexico, this is one of the main sources of exposure. No study discussed or

			mentioned cultural aspects of lead exposure.
	-"the form of sampling makes the authors finding of an increase odds...even more remarkable": Please clarify the reasoning behind why this is remarkable. Selection bias on the basis of self-selection into participation can drive effect estimates in any direction based on what reasons people choose to participate. For example, those from poorer neighborhoods (with higher lead) and more antisocial behavior may choose to participate leading to this observation.	N/A	This paragraph has been rephrased for clarity. Selection bias was one of the categories assessed through the ROBINS-E tool, and the mentioned study was categorized as “serious” for selection bias.
	"the authors [Dietrich study] address this and state this finding [may] be due to..." not sure how this statement is supported. Understanding this is a paraphrase of the original paper, do the current authors think it's justified?	Lines 460-462	This section has been rewritten to clarify the statement.
	-"the authors chose to incorporate ecological as well as individual level data...may obscure the true impact of lead exposure": How would it obscure if effect sizes of ecological studies are orders of magnitude higher? Does this sentence just mean the estimates are biased? I believe Higney, et al make it clear that those effects are likely biased and give a much smaller estimate of effect in their meta-analysis. Can you clarify this statement, otherwise it appears you are misrepresenting those authors' findings.	Lines 469-475	This section has been rewritten for clarity, and a statement to the section “consistency with the existing literature” has been added to address this.
**Reviewer 5**	1. A topic as sensitive as this one requires a great deal of care and precision with language. The primary research question emphasizes “crime” but then a much wider array of outcomes is included and discussed (e.g. “aggression”, “anti-social behavior”, “rule-breaking behavior”). Furthermore, some of these terms are not defined, for example “indirect aggression.” I would recommend reviewing the language	Lines 465 - 470	Explicit definitions of “indirect aggression” and “direct aggression” were added to clarify this point.

	used to ensure it is consistent and accurately reflects what is ultimately emphasized in the manuscript.		
	2. While it is encouraging that there were eligible studies from Brazil and South Africa, a slightly expanded discussion on the geographic limitations of this review is warranted. This could include the lack of any low-income countries, no representation from Asia, and importantly, an overrepresentation from the US (8 of the 17 studies were from the US, and 3 from just one city).	Lines 426 - 429	Our review was conducted with the help of a librarian and encompassed all studies on a global scale available in any language that pertained to aggressive and criminal behaviors and lead exposures. We did not intentionally exclude studies from within Asia, there simply were none to analyze per the parameters of the review. In our updated draft, we have made a strong recommendation encouraging further research within this field on a more diversified, global scale.
	It appears that there is an overlap in the categories for “age at exposure measure” (<6, 5-6, 6-11). This becomes particularly confusing in the “General Interpretation” section, where the prenatal and 5-6 year period are highlighted as being of particular concern regarding negative outcomes.	Table 3	There is an overlap in categories with the Cincinnati cohort as they analyzed a window of exposure from 5 - 6.5 yo. We decided to include it as early childhood because we felt it was more appropriate. A note has been made to Table 3 to mention that.
	4. In the bias assessment, it is troubling that 7 of the 17 studies have serious or critical concerns about confounding. As the authors note, factors like socioeconomic status and race are hugely influential on lead exposure levels, and independently, on outcome variables like arrest rates. The authors could examine how excluding the studies found to have a high-level of bias (either for particular bias categories or the overall bias metric) influences the conclusions.	Lines 581 - 584	Results from the ROB have been included in Table 3 for better visualization. We increased the discussion on how excluding studies in the category “serious” could influence the conclusions.

	1. There are some inconsistencies in the reporting of the numbers of studies. For example, in the abstract, it is reported that there are 14 BLL studies, 3 bone lead, 1 dentine, but these numbers are different in Table 2. In line 201, it states that 9 studies are from the US but Table 2 indicates 8.	Table 2	Thank you for that note, this has been fixed in table 2.
	2. I would recommend some acknowledgement of the fact that bone and dentine lead reflect cumulative exposure, while blood lead provides a snapshot of recent exposure.	Lines 235 - 238	This can now be found in section: Study Characteristics: Exposure Status.
	3. Line 45. "individuals" rather than "communities" may be more appropriate here, as the references cited do not appear to reflect population level trends in these health outcomes.	Line 46	This has been changed in the Introduction section.
	4. Line 292. Refers to "African Americans" but one of these studies is from South Africa.	Lines 277-278	This has been fixed and only includes now studies that included African-Americans (US studies)
	5. Section "Adolescence and adult exposure" (no page numbers) - typo "studid"	Line 361	Thank you, this typo has been fixed.
	6. Section "Trends in evidence" - 1st sentence. I believe this refers to the severity or magnitude of lead exposure, not "rates"	Line 407	Thank you, this has been changed
	7. Section "Augmentation of the ROBINS-E Tool: Included Confounders" - African Americans are referenced explicitly again here, but as this paper is international in scope, are there other relevant trends regarding lead exposure and race identified in other countries?	Lines 559 - 560	We did not require race to be a confounder for countries that sampled from a homogenous racial distribution. This is now explicitly mentioned.
