# Supplementary material for: The association between lead exposure and crime: A systematic review
Source: PLOS Glob Public Health. 2023 Aug 1;3(8):e0002177. doi: 10.1371/journal.pgph.0002177 (PMC10393136; doi:10.1371/journal.pgph.0002177)
Supplement: Attachment — Submitted filename: Response to Reviewers May 2023.pdf [file pgph.0002177.s007.pdf]

Milken Institute School of Public Health
THE GEORGE WASHINGTON UNIVERSITY

Dr. Maria Jose Talayero Schettino
Department of Environmental and Occupational Health
The George Washington University
950 New Hampshire Avenue
Washington, DC, 20052
(917) 972-4571
mjtalayero@gwmail.gwu.edu

Dr. Naveen Puttaswamy, Ph.D., Academic Editor
Dr. Jonathan Huang and Associates, Peer Reviewers
Journal of PLOS Global Public Health
1265 Battery Street, Suite 200
San Francisco, CA
94111 USA
(415) 624-1200
em@editorialmanager.com

May 30, 2023

Dear Drs. Puttaswamy, Huang, and Associates,

Thank you for your letter regarding our manuscript entitled "The association between lead exposure and crime: a systematic review." We appreciate your consideration and time regarding this manuscript, and we think you will find that the revised and updated version is appropriate for publication in *PLOS Global Public Health.*

Our team thoroughly reviewed each comment, and we are grateful for such detailed and thoughtful feedback. We have attached a detailed table to this letter with each individual comment addressed. All of the suggestions and corrections made by the reviewers were implemented into the latest draft, with thanks for the thoughtful comments.

We feel that the peer review process has added to the strength of our paper and look forward to your further feedback and review. Please do not hesitate to contact us if there are any further questions.

With thanks for your time and assistance,

Dr. Maria Jose Talayero

Reviewer	Comment	Line Addressed	Comment from authors
**Reviewer 5**	I would suggest you indicate that this is specific to the US, or else include non-US references. Does this trend hold true in other countries (South Africa?)	557	Thank you for this comment, we agree, the statement was rewritten to show it is US specific.
	How was it determined whether the studies sampled from a homogenous racial distribution? Was race reported for all included studies (but not necessarily included as a confounder)?	556 - 562	While the majority of studies reported race as a potential confounding factor, not all studies included it in their analysis. In cases where race is not uniformly distributed within a country (e.g., the US, New Zealand), we specifically requested its inclusion as a confounder, as outlined in Supplementary Table III. However, for studies conducted in African settings, where authors reported uniform racial distributions within the cohort we did not mandate the inclusion of race as a confounding variable in our assessment of risk of bias. The paragraph has been rewritten for clarity.
	If you report the numeric change in aggression score, perhaps include the scale of the test for context.	373	The scale was added for context.
	This is the first time shooters is mentioned – explained later as “gun range participants” (440)	380 and 440	Thank you for your comment, the statement has been rewritten for consistency. “Gun range participants” is now used.
	I would recommend rewording this as “individuals with high levels of dentine lead” (the dentine lead is the result of exposure, not the source of it)	434	This line has been rewritten for clarity as suggested.
	Microgram/dL (not milligram). Also, sometimes mcg/dL is used and sometimes µg/dL throughout manuscript	440 - 441	A thorough revision of the manuscript was conducted and all units were changed to ug/dl for consistency.
	What does the word “sensitive” here mean? That the negative effects of a	535 - 537	The statement was confusing, it has now been rewritten for clarity

	comparable lead exposure level are more pronounced in a lower SES child than a higher SES child?		as follows: It has been well documented that children from economically disadvantaged households tend to exhibit heightened susceptibility to the various detrimental effects caused by lead exposure
	Please define/explain HOME acronym	547 - 548	The HOME acronym has been defined: Home Observation for Measurement of the Environment
	While I completely agree that cultural factors such as use of lead-glazed ceramics would impact the magnitude of lead exposure in an individual, it is not clear to me how such factors would influence the relationship between lead exposure and crime/antisocial behavior.	N/A	Thank you for your comment, we agree that it should not be included in the confounder section, so it has been moved to the sources of exposure paragraph at the beginning of the manuscript.
	What is implied by “unconventional parenting”? This seems vague and very subjective.	593	We agree, the term is subjected and has been deleted from the manuscript
